# Patient and healthcare professionals’ perception of weekly prophylactic catheter washout in adults living with long-term catheters: qualitative study of the CATHETER II trial

**DOI:** 10.1136/bmjopen-2024-087206

**Published:** 2025-04-07

**Authors:** Sheela Tripathee, Mohamed Abdel-Fattah, Diana Johnson, Lynda Constable, Seonaidh Cotton, David Cooper, Graeme MacLennan, Suzanne Evans, Amanda Young, Konstantinos Dimitropoulos, Hashim Hashim, Mary Kilonzo, James Hugh Larcombe, Paul Little, Peter Murchie, Phyo Kyaw Myint, James NDow, Catherine Paterson, Karen Powell, Graham Scotland, Nikesh Thiruchelvam, John Norrie, Muhammad Imran Omar, Sara J Maclennan

**Affiliations:** 1 Academic Urology Unit, University of Aberdeen, Aberdeen, UK; 2 Aberdeen Centre for Women’s Health Research, Institute for Applied Health Sciences, 2nd Floor, Aberdeen Maternity Hospital, Foresterhill, University of Aberdeen, Aberdeen, UK; 3 Centre for Healthcare Randomised Trials, Health Services Research Unit, University of Aberdeen Institute of Applied Health Sciences, Aberdeen, UK; 4 Centre for Healthcare Randomised Trials, Health Services Research Unit, University of Aberdeen, 3rd Floor, Health Sciences Building, Foresterhill, University of Aberdeen Institute of Applied Health Sciences, Aberdeen, UK; 5 Health Services Research Unit, Centre for Healthcare Randomised Trials, University of Aberdeen, Aberdeen, UK; 6 Centre for Healthcare Randomised Trials, Health Sciences Building Foresterhill, University of Aberdeen Institute of Applied Health Sciences, Aberdeen, UK; 7 Bladder Health UK, Birmingham, UK; 8 Queen’s Nursing Institute, London, UK; 9 Department of Urology, Aberdeen Royal Infirmary, Aberdeen, UK; 10 Bristol Urology Institute, North Bristol NHS Trust, Westbury on Trym, Bristol, UK; 11 Health Economics Research Unit, University of Aberdeen, Aberdeen, UK; 12 NHS Durham Dales Easington and Sedgefield CCG, Sedgefield, County Durham, UK; 13 University of Southampton, Medical School, Southampton, UK; 14 Academic Primary Care, University of Aberdeen, Aberdeen, UK; 15 Institute of Applied Health Sciences, University of Aberdeen Institute of Applied Health Sciences, Aberdeen, UK; 16 Department of Surgery, University of Aberdeen, Aberdeen, UK; 17 School of Nursing, Midwifery and Public Health, University of Canberra, Canberra, Australian Capital Territory, Australia; 18 Obstetrics and Gynaecology, University of Aberdeen, Aberdeen, UK; 19 Cambridge University Hospitals NHS Foundation Trust, Cambridge, UK; 20 Edinburgh Clinical Trials Unit (ECTU), Edinburgh Royal Infirmary, Edinburgh, UK; 21 Guidelines Office, European Association of Urology, Arnhem, The Netherlands

**Keywords:** clinical trial, qualitative research, self-management, health services, urology, urinary incontinences

## Abstract

**Objectives:**

To explore trial participants’ experience of long-term catheters (LTC), the acceptability of washout policies, their experience of the CATHETER II trial (a randomised controlled trial comparing the clinical effectiveness of various washout policies versus no washout policy in preventing catheter associated complications in adults living with long-term catheters) and their satisfaction with the outcomes. The objectives of the healthcare professionals (HCPs) focus group and interview were to explore their attitudes towards weekly prophylactic catheter washout, views on the provision of training and participants’ ability to enact washout behaviours.

**Methodology:**

A longitudinal qualitative study embedded within the CATHETER II randomised controlled trial, which included semi-structured interviews and focus groups with participants from multiple trial sites. Data were analysed using the Theoretical Framework of Acceptability and Theoretical Domains Framework. This UK community-based study included 50 (24 female, 26 male) CATHETER II trial participants, aged between 23 and 100 years, with LTC and able to self-manage the washout and study documentation either independently or with the help of a carer. Seven HCPs (five female, two male) also participated.

**Results:**

The participants had positive attitudes towards weekly prophylactic saline or acidic catheter washouts and other trial elements, such as washout training, catheter calendar and monthly phone calls. Participants and HCPs found the ‘ask’ of the CATHETER II trial and the weekly self-administered prophylactic washout policies to be feasible. The participants reported that the catheter washout training provided during the trial enhanced their self-efficacy, skills and self-reported capability to carry out the washouts. Participants reported having positive outcomes from the weekly washout. These included reduced blockage, pain or infection, reduced need for HCP support and greater psychological reassurance. HCPs attested to the participants’ understanding of and adherence to the weekly washouts and other elements of the trial.

**Conclusions:**

This study shows acceptability, feasibility and self-reported fidelity of the CATHETER II trial on a behavioural level. Self-management for prophylactic catheter washouts is both feasible and, following training, achievable without any need for additional support.

**Trial registration number:**

ISRCTN17116445.

**STRENGTHS AND LIMITATIONS OF THIS STUDY**

- To our knowledge, this is the first qualitative study embedded within a randomised controlled trial to report on patient and healthcare professionals’ perceptions of prophylactic washout.
- Longitudinal data from patients prior to and after taking part in the trial and theory-based analysis provide rigour to the interpretation of the findings.
- A combination of an inductive and deductive approach for the analysis, and use of the theoretical frameworks ensured that contextual nuances were carefully considered in generating the findings and meta-themes in this study.
- Despite the difficulty in recruiting trial participants during and after COVID-19, the participants in the qualitative study represent 62% of the total CATHETER II trial participants from 76% of the recruiting sites.
- The number of healthcare professional participants within the embedded qualitative study is lower than we initially anticipated; however, these views are representative of six out of the 21 recruiting sites, and the concordance in views provides confidence in the findings.
- The patients in the study were the ones already engaged in the CATHETER II trial; therefore, their views may not be representative of those who were less inclined to engage in washout behaviour and hence did not take part in the CATHETER II study.

## Introduction

 People with urinary incontinence and/or urinary retention^1^ often use urinary catheters. In the UK, approximately one in 700 people use a long-term catheter (LTC).^2^ LTC use is expected to continue to increase with the ageing population due to many more people living with multiple long-term conditions necessitating the use of an LTC.^2^ However, LTC is associated with various complications, including LTC blockage, pain, bladder spasm, symptomatic catheter-associated urinary tract infection (S-CAUTI) and urinary leakage.^3 4^ Around half of all people living with LTC are affected by LTC blockages.^5^ Catheter blockages may cause significant pain, anxiety and incontinence, which have a profound negative impact on quality of life.^6^ These complications also increase patients’ reliance on health resources, such as out-of-hours nursing visits, repeated visits to primary care and/or hospital admissions, adding to the burden on National Health Service (NHS) resources.^7^ LTC encrustations and blockages are commonly managed by using liquid washout (saline or acidic) solutions to flush the catheter.^8 9^ However, prophylactic washouts are not recommended in current guidelines to prevent blockages. Evidence on benefits or potential harms associated with prophylactic washout practice with either saline or acidic liquids to prevent blockages and other LTC-related adverse events compared with no washout policy is reported to be of low quality and insufficient.^8^,^11^

CATHETER II was a three-arm multicentre randomised controlled trial (RCT) that aimed to investigate the clinical and cost-effectiveness of a policy of weekly prophylactic LTC washouts with either saline or citric acid, in addition to standard LTC care, compared with standard LTC care only in adults living with LTC.^12 13^ The details of the CATHETER II trial and outcomes are reported in two separate publications,^12 14^ which suggest lower rates of LTC blockages without increasing S-CAUTI when employing prophylactic LTC washout. This paper presents findings from the embedded qualitative study, referred to hereafter as the CATHETER II qualitative study.

Qualitative research embedded within an RCT provides a better understanding of the context, feasibility, acceptability and usefulness of the intervention.^11^ This approach can be particularly useful to gain a comprehensive insight into the effects of complex healthcare interventions involving social or behavioural processes that are difficult to evaluate using only quantitative methods alone.^15^ Understanding participants’ views on, and experience of, the trial may also help strengthen the implementation of the trial and inform policy around the effective LTC washout. Participants’ and healthcare professionals’ (HCPs) perceptions on, and experience of, various catheter washout policies within the context of an RCT have not previously been explored.

The CATHETER II qualitative study aimed to explore participants’ experience of LTC, their acceptability of washout policies, their experience of the CATHETER II trial including washout training and satisfaction with their experience and the HCPs’ perceptions of the training provision and enactment of participants’ treatment skills, abilities and attitudes towards washout policies.

## Methods

This qualitative descriptive study^16^ has been reported according to Consolidated criteria for Reporting Qualitative research.^17^ online supplemental file 2

### Recruitment

CATHETER II trial participants (randomly allocated to saline washout group, citric acid washout group and standard LTC care group) who consented to be contacted for the qualitative study were sent a qualitative participant information sheet and consent form. Consenting participants were contacted by the qualitative research team to respond to any queries, confirm participation and schedule the interview.^13^ Maximum variation sampling was used to include participants from all trial arms, genders and ages to maximise the potential for diverse perspectives. Sample size was planned by following the principles of Information Power.^18^ The key features of this study were: a relatively narrow aim and focus, dense sample within each arm of the trial, interviews to be conducted with the same participants at two time points, analysis to be guided by established theories and a strong research team with expertise that would ensure high-quality conversation with participants and additional data from HCPs. Considering these features, a sample size of 40 patients, proportionally distributed across the three arms of the trial was deemed adequate and sufficient for descriptive and interpretive depth to ensure that the theoretical underpinnings were clearly articulated and the study objectives were met effectively.

All HCPs involved in the CATHETER II trial were given information about the qualitative component and invited to take part in focus groups or for interview, and all interested HCPs were included.

### Data generation and management

Data were collected from February 2020 to August 2023. During and after the COVID-19 pandemic, both interview and focus group participants were contacted and interviewed virtually using telephone or Microsoft TEAMS.

Semi-structured interviews were conducted with 40 participants at two time points: the first interview (T1) was conducted prior to the participants’ knowledge of which study arm they were randomised to, and the follow-up interview (T2) was conducted 6–12 months later. Ten participants from the T1 cohort were unavailable for follow-up interview at T2. Therefore, an additional 10 participants with similar characteristics were recruited and interviewed for T2 data collection.

Focus group and interviews were conducted with seven HCPs at least 6 months after the study commenced. The focus group was conducted with HCPs involved in the trial from six of the 21 sites. Due to the small cohort of HCPs in the trial who consented to take part in the qualitative study, only one focus group was conducted. One further HCP interview was conducted to check the data and ensure the robustness of the focus group data. The participants’ views within the focus group and the data from HCP interviews were consistent, and no new information emerged from the interview. Therefore, no further interviews were conducted, and the data were considered adequate.

Topic guides (online supplemental table 1) for the interviews and focus group were developed following discussions with the study team, review of the scientific literature^8 9^ and existing patient interviews on www.healthtalk.org.^6^ The topic guides were piloted with the public and patient representatives and refined. The topic guide for the T1 interviews included questions on participants’ LTC-related complications, such as blockage, urinary incontinence and bladder pain, their attitudes and catheter washout preference and expected outcomes. The topic guide for the follow-up (T2) interviews included questions on experience of, and satisfaction with, the trial process, washout training and self-management skills and outcomes related to LTC management and complications. The HCP focus group and semi-structured interviews explored attitudes towards washout policies, perception and experience of the CATHETER II trial, and views on likely outcomes.

A female researcher (ST) with a PhD in Health Sciences and expertise in qualitative research methods conducted all the interviews and focus groups. The majority of the interviews were conducted with participants alone; however, three participants preferred and consented for their carer to answer the interview questions on their behalf in their presence. During the interviews and focus group, participants were encouraged to discuss any relevant issues important to them beyond the topic guide. Reflective notes were taken during the interviews/focus group to facilitate increased transparency and trustworthiness of the data and were used to help support decision-making in the analysis. The interviews and focus group were recorded using a digital recorder and professionally transcribed verbatim. All transcripts were checked for accuracy and anonymised by removing all identifiable information and replacing the names with pseudonyms. NVivo (V.12 for Windows, QSR International, https://www.qsrinternational.com/nvivo-qualitative-data-analysis-software/support-services) was used to organise the data and support analysis.

### Data analysis

All interviews and focus group transcripts were analysed using a structured qualitative method, Framework analysis approach, guided by the Theoretical Domains Framework (TDF) and the Theoretical Framework of Acceptability (TFA). The TDF is an integrated theoretical framework that guides content analysis of qualitative data within defined domains of behaviour change such as knowledge, skills, self-confidence, optimism, intentions, goals, memory and attention, behavioural regulations and environmental and social context. It helps to rigorously identify the behavioural processes or barriers and facilitators involved in behaviour change and assess implementation processes.^19^,^22^ TFA provides a comprehensive guide for the assessment of acceptability of healthcare interventions and is used to inform a robust understanding of acceptability across core constructs such as intervention coherence, self-efficacy, perceived effectiveness, attitude and burden.^23 24^

The interview and focus group data were coded both inductively and deductively within TDF and TFA domains. After the first reading of the transcripts, initial themes related to each TFA and/or TDF domain were identified. Any other statements relevant to the study but not within the scope of the TFA or TDF domains were coded inductively, including participants’ experience of LTC-related complications prior to the trial. Statements relevant to multiple TDF and TFA codes were included under all the relevant codes.

To enrich rigour, randomly selected six transcripts and thematic outputs emerging from the interviews were read by two researchers (ST and SJM), and the transcripts were deemed consistent. Any circumstantial inconsistencies were discussed among the research team. Multiple coding by ST and SJM tested the reliability of the designated codes (see online supplemental table 2).

After the initial coding, domains addressing common related research objectives were grouped into themes, and frameworks were generated. The research team discussed these frameworks and themes and generated belief statements (representative description of the statements within the domains). Any conflict in interpretations was discussed between the researchers, and themes were redefined accordingly. Frameworks were created to map the complete dataset to identify patterns within and across arms of the trial, and participant profiles according to the study aims. This process helped identify themes and subthemes across all interviews and domains and examined any potential interconnected relationships. HCP focus group and interview data were initially coded separately and analysed, then triangulated with the participants’ interview data to identify key common themes for final analysis.

### Patient and public involvement

Patient and public involvement (PPI) representatives were actively involved in all stages of the study. They were members of the project management group, trial steering committee and involved in running and reporting of the study. Patient interviews (on www.healthtalk.org) were reviewed to design the study and develop the topic guides. Recruitment documents and data topic guides were piloted with and reviewed by patients. PPI partners will be involved in disseminating the findings to participants and the public.

## Results

### Participant profile

A total of 50 (24 female and 26 male) trial participants aged 23–100 years from 16 CATHETER II recruitment sites across the UK were included (tables1  2). Eleven participants had been living with LTC for less than a year, 16 for 1–3 years and 23 for >3 years. Seven HCPs (five female and two male) participated from six sites across the UK. Six HCPs participated in a focus group and one HCP was interviewed. HCPs included one urogynaecologist and six research nurses with varying nursing backgrounds (community, district, primary care or continence). The length of the interviews ranged from 22 to 58 min and the focus group was 90 min long.

**Table 1:** Participant characteristics

Pseudonyms	Arm	Gender	Age (years)	LTC duration (years)
Anderson	3	M	71–80	1–3
Bruce*	2	M	81–90	<1
Caroline	1	F	61–70	<1
David*	2	M	71–80	>3
Elizabeth	1	F	20–30	>3
Emily*	2	F	61–70	>3
Freya*	3	F	31–40	>3
Gorden	2	M	71–80	>3
Haward	1	M	61–70	<1
Hazel	2	F	61–70	1–3
Heather†	3	F	20–30	<1
Jackson	3	M	81–90	<1
Jean	3	F	51–60	>3
John	3	M	91–100	<1
Karen	2	F	71–80	>3
Keith	3	M	61–70	1–3
Kenny	3	M	71–80	1–3
Kevin†	1	M	71–80	1–3
Kirsty†	1	F	51–60	>3
Lorna*	3	F	61–70	<1
Lorraine	3	F	61–70	>3
Lou	2	M	61–70	>3
Loutomia†	2	F	61–70	>3
Mark*	2	M	61–70	1–3
Mark*	2	M	71–80	1–3
Mathew†	3	M	91–100	>3
Melissa*	2	F	20–30	<1
Monica	3	F	61–70	>3
Paul	1	M	61–70	1–3
Paul†	2	M	71–80	>3
Pauline	3	F	61–70	>3
Peter	3	M	71–80	>3
Philip	1	M	71–80	<1
Rachel	1	F	61–70	1–3
Ralph	2	M	71–80	>3
Richard	2	M	71–80	1–3
Ruth	1	F	61–70	>3
Ruth*	2	F	61–70	>3
Sandy	2	M	71–80	>3
Shaun†	1	M	71–80	1–3
Stella	1	F	71–80	1–3
Stephanie	1	F	41–50	1–3
Stephen	3	M	51–60	>3
Steve†	2	M	51–60	<1
Susan†	2	F	51–60	>3
Tailor	3	M	61–70	>3
Virginia	1	F	71–80	1–3
Wendy	3	F	41–50	1–3
William†	2	M	61–70	<1
Winston*	1	M	91–100	1–3

Arm: 1=saline washout group (sa), 2=citric acid washout group (ca), 3=standard LTC care group (uc).

* Only interviewed at T1.

† Only interviewed at T2.

FfemaleLTClong-term catheterMmale

**Table 2:** Summary of participants’ characteristics

Arm 1 (sa): allocated standard LTC care+saline washoutN=14	Arm 2 (ca): allocated standard LTC care+citric acid washoutN=19	Arm 3 (uc): allocated standard LTC care (no washout)N=17
Female=9, male=5	Female=7, male=12	Female=8, male=9
T1 interviews=11T2 interviews=13	T1 interviews=14T2 interviews=12	T1 interviews=15T2 interviews=15
Interviewed only at T1=1Interviewed only at T2=3	Interviewed only at T1=7Interviewed only at T2=5	Interviewed only at T1=2Interviewed only at T2=2
Moved from citric acid to saline washout=1		Moved from saline washout to standard LTC care=1
Duration of LTC<1 year=31–3 years=8>3 years=3	Duration of LTC<1 year=41–3 years=4>3 years=11	Duration of LTC<1 year=41–3 years=4>3 years=9

cacitric acid washout groupLTClong-term cathetersasaline washout groupucstandard LTC care group

A thorough exploration and analysis of trial participants’ and HCPs’ perspectives provided insights around acceptability, feasibility and fidelity of the CATHETER II trial (eg, weekly catheter washout behaviour) that were explained by participants’ experience prior to the trial, their knowledge, attitude, intervention coherence, self-efficacy, optimism, environmental context and resources and perception of skills, capability, burden, effectiveness and consequences. These dominant theoretical domains were summarised within three meta-themes: “I like the idea of washout” (acceptability of weekly washout behaviour and CATHETER II trial); “it’s quite straightforward once it’s sort of explained to you” (feasibility of weekly washout behaviour and CATHETER II trial) and “I do it myself … each week” (fidelity of weekly washout behaviour and CATHETER II trial). Illustrative quotes that are not included in the ‘Results’ section are presented in a table of themed quotations in online supplemental table 3.

### “…I like the idea of washout…” (acceptability of weekly washout behaviour and CATHETER II trial)

Participants’ engagement with the CATHETER II trial and their views on the acceptability of and commitment to the weekly catheter washout were explained by their previous LTC experience, affective attitude, intervention coherence, optimism**,** perceived effectiveness and belief about lack of negative consequences. All participants were aware of the purpose of the trial, the different trial arms, the duration of the trial, the randomisation process and the potential commitment needed from them to take part based on the trial arm they could be randomly allocated to.

### Positive affective attitude and perceived effectiveness

At T1, many participants described being unaware of catheter washout options and/or practice prior to receiving the trial information. Most of these participants reported that district nurses were their point of contact for catheter management (routine change) as well as for complications, such as blockage. Catheter change was reported as a common practice in case of blockage. Only a few described attempts to treat the blockage with washout. Most participants reported having a prior complication related to their LTC, such as blockage, S-CAUTI, urinary incontinence or bladder pain requiring hospital care for their catheter-related complications. Many of these participants described the trial as an opportunity to learn more about their catheter management and washout.

I didn’t know much about the catheters and what the difference is with each one (washout), … I mean I’ve never spoken to anybody that has one so … I just want to learn as much as I can about it. Pauline 61–70, uc.

Most participants expressed positive attitudes towards catheter washout. They believed/expected that weekly washout would be beneficial, with only some participants being unsure of potential benefits. These expected benefits related to reduced infection, reduced or no blockage, reduced pain/spasm and a clean catheter from weekly washout.

I prefer the washout because hopefully that would prevent me getting any blockages. Mark 61–70, ca

### Perceived lack of negative consequences, opportunity cost or burden

Participants’ optimism and lack of concern about potential harm also appeared to be a notable reason for their interest in taking part in the trial. Many participants did not expect any side effects and only a few expected some irritation or increased chance of infection from washout or expressed uncertainty about potential side effects. Trial arm preference was mixed, with reports of no preference, as well as preference for either the citric acid washout or the saline washout. Only one participant expressed a preference for the standard LTC care group with no washout.

Well, it’s choosing something you’ve never experienced … I like the idea of the washout. It seems to me that to wash the thing out on a weekly basis would well-prevent the thing from blocking. Gorden 71–80, ca

### Contribution to knowledge

In addition to the expected benefit of the potential weekly washout to their catheter management, most participants described that a key motivation for taking part in the trial was altruistic. In particular, for those with no prior LTC-related complications, contribution to research was their primary motivation for participating in the trial.

Yes, it could help people in the future and of course it might help myself as well. Stephen 51–60, uc

A few participants specifically emphasised that the trial could help them and others by potentially eliminating the variation in washout policies.

I just think that it will be good for there to be perhaps a standard practice across the board rather than some areas liking washouts and some areas not doing washouts. Freya 31–40, uc

There were no notable differences in participants’ descriptions of their prior LTC experience, attitude, expectation and potential barriers and facilitators to their participation between different groups (eg, arm of trial, gender or age).

### HCPs’ perception of evidence need for policy and practice

HCPs involved in the trial at local recruiting sites described being very happy to be part of the trial and found their roles within the trial to be appropriate and important.

I was really keen and really happy to take part and as were my clinical colleagues as well. Jennifer, HCP3

HCPs expressed unanimous views about the need for the CATHETER II trial due to the lack of evidence for washout policies and inconsistencies in washout practices across the UK. HCPs highlighted that there was no robust evidence on the effectiveness or disadvantages of prophylactic washouts, and if a particular type of washout solution was better than the other. Therefore, HCPs believed that the evidence produced from the trial was important for evidence-based policy development clinical practice, making the best use of health resources and improving patient experience.

Everything that we are doing is based on anecdotal evidence… you cannot really base a decision on the current available evidence […] At the moment, there is no recommendation that there should be any prophylactic washouts using any solutions and that’s not a reflection on evidence but it is a reflection on the fact of the lack of evidence. Lawrence, HCP7

We don’t know whether what we’re doing is good or bad or whether it’s useful at all beyond the anecdote. Keith, HCP2

Some HCPs expressed concerns over the variation in practices across the country and that their current practice may not provide the best outcomes for patients. They described the evidence produced from the trial would determine whether or not washout practices are effective and would be a key in establishing best practices and efficient use of resources for themselves and for the NHS.

The evidence either way will save us time, money, resources. If it’s found that they are effective and that we should be doing them routinely, then that might save us time with going out and changing catheters and these emergency visits … from a patient point of view if you’re having to call a nurse out every week because your catheter’s blocking but there’s a solution to that then again, that’s going to help them too. Jennifer, HCP3

### “…it’s quite straightforward once it’s sort of explain to you…” (feasibility of weekly washout behaviour and CATHETER II trial)

#### Perceived ability

Participants’ skills, belief about capability, optimism, intervention coherence, perceived self-efficacy and environmental context and resources explained their confidence in their ability to carry out the weekly washout even prior to taking part in the trial.

At the T1 interview, almost all the participants expressed their belief about capability and optimism that they (or their carer) would be able to carry out the weekly washout and other trial tasks. While many suggested their lack of skills and awareness of conducting washouts as potential barriers, they were cautiously optimistic that the washout training to be provided during the trial would equip them with the necessary skills. Many expected to be able to fully participate in the trial without any support, a few expected to be able to carry out the washout and other trial tasks by themselves with support and some said their carer for their routine catheter management would conduct the washout and other trial tasks. Some described having this confidence due to prior washout experience or knowledge, and the rest were confident that the training and support from trial nurses would be sufficient for them to learn about and carry out the washouts. Previous experience of managing their catheter in general underpinned their optimism.

Once I’m shown how to do something, I’d be able to do it. Like I said, I change the leg bag every week. Richard 71–80, ca

If I do get one of the washouts training I can carry it on. Anderson 71–80, uc

The data from T2 participants’ interviews and HCP focus group showed that participants’ self-efficacy, skills, belief about capability, perceived effectiveness, optimism, (lack of) burden and opportunity cost domains explained their enactment of the washout behaviour and satisfaction with the trial.

Although many had alluded to their lack of skills or experience and added caution to their optimism about their ability at T1, at T2 almost all described that the increased confidence and skills they gained during the trial facilitated their ability to carry out the weekly washouts. Many were able to conduct the weekly washouts by themselves or with someone’s support and only two participants described not being able to manage the washouts successfully. Those able to carry out weekly washouts and other aspects of the trial, such as filling in the catheter calendar (a form of diary) and monthly phone calls said they did it without any additional support beyond their usual catheter care. Most participants reported being able to complete all the trial tasks as required.

It takes me about 30 minutes for the whole process … it’s straightforward … it’s certainly got easier with time. Paul 71–80, ca

It was good, it was quite straightforward once it's sort of explained to you and as I say, it takes a little bit of getting used to but once you’ve done it a few times then it’s more easy to do. Stephanie 41–50, sa

#### Intervention coherence, self-efficacy and resources needed

When discussing the acceptability and feasibility of the CATHETER II, most of this dialogue was grounded in intervention coherence, perceived self-efficacy to self-manage and the resources needed to support this. The focus was centred on the training provided and the trial tools (eg, catheter calendar).

The training provided to all participants in the washout groups at the start of the trial was described by most as essential for increasing self-efficacy and capability to self-manage, and as effective and easy to follow. For most participants, one training session was sufficient. A few were not confident to carry out the washouts after the initial training and were offered additional training sessions or follow-up calls. Only two participants in the washout groups described being unable to carry out the washouts successfully even after receiving training.

Nurse showed us how to do it … It was very helpful … it was fine just at once. Caroline 61–70, sa

There was a video to watch and some instruction from the hospital, from the nurse I spoke to … Well I had no problem with that although initially I wasn’t doing it correctly but that was resolved quite quickly with help over the telephone and then I found it was okay. Philip 71–80, sa

There were no notable differences in participants’ description of their experience of the training or weekly washout behaviour and its outcome between saline and citric acid washout groups. Most described the trial and their role as being in line with their pre-trial perception and expectation.

Most participants said that they were able and happy to complete the catheter calendar, questionnaires and monthly phone calls. While some of the participants in the standard LTC care group expressed disappointment at not being included in one of the washout groups, most of them described the calendar and monthly phone call as straightforward, and they were able to complete the calendar and provide information by themselves or with the help of a carer.

I will definitely complete the trial because it’s a really interesting trial to be in. I was a bit gutted that I didn’t get the washout, but it hasn’t affected me being in the trial. Heather 20–30, uc

Regardless of the impact of the prophylactic washout on their LTC management or related complications, most participants reported increased confidence in their ability to self-manage their catheter and/or washout as a result of the trial.

Similar to the participants’ accounts, all HCPs involved in the trial delivery described the training as essential and effective for patient self-efficacy and self-management, and appropriate and not too onerous on HCPs. They stated that the participants responded well to both virtual and in-person training. The flexibility of offering either in-person or virtual training was viewed as important as a few participants needed in-person training due to limitations of their IT skills. The availability of video instructions was believed to be beneficial for many. HCPs used the training for participants with LTC outside the trial as well and stated that participants felt empowered and capable of self-management, which is beneficial for both participants and healthcare systems in supporting the sustainable care of LTC.

It was easy, the patients followed the instructions. Jill, HCP6

We’ve kind of used that to educate others how to do their own washouts as well even if they’ve not wanted to go on the trial. Susan, HCP1

Consistent with the participants’ views, HCPs also described the participants as being interested and highly engaged during the trial. Participants’ skills and abilities to successfully carry out catheter washout were also confirmed by the HCPs. HCPs involved in the trial reported that participants were confident and able to carry out weekly washouts themselves or with support from their usual catheter management carer following the initial training and did not require any additional support. HCPs also highlighted that regardless of the evidence on prophylactic washout policy, providing participants with the washout training could be beneficial as it empowers them to have the confidence and skills to self-manage multiple LTC-related complications.

They had turned their own mind to managing their own catheter rather than it being this foreign object that is just planted in that somebody else come and deals with […] most of them can do it themselves as has been seen on this trial, and all you need is one person to be trained to teach them to do it … it’s not necessarily a big onus of time for teams. Keith, HCP2

It proves they can do it and they can take a bit of control, as long as they’ve got someone at the end of a phone that they can ask questions of when they need to. Jill, HCP6

HCPs reported that all participants, including those within the standard care control arm who did not receive weekly washout, could complete the trial tasks after receiving the initial instructions and said that the catheter calendar was a useful tool that provided them with confidence in their catheter management and supported care providers.

It [catheter calendar] might be worth giving to people who are starting to experience problems with their catheters so that you can document exactly where the problem lies and then maybe get more of an early intervention rather than having to call a district nurse out when actually the catheter is already blocked. Jennifer, HCP3

It’s sort of made them more aware of things. Wendy, HCP4

The CATHETER II trial design, the high standard of trial management, the empowerment of participants with self-management skills and tools and no significant burden on the healthcare system were described as key virtues of the trial.

It was designed in a way that it would be self-care with the washouts because that means that you do not have to add a lot of workload for the already stretched district nurses. Lawrence, HCP7

To be able to do the training remotely meant that they didn’t have to come to hospital, and a lot of people like that because often they wouldn’t have access to some of these health services otherwise. Ruth, HCP5

For a small number of cases, HCPs found a lack of interest in patients who declined to take part in the trial. HCPs described that some eligible patients might have declined participation due to their misconception that they may be unable to carry out washouts if they were allocated to the standard care arm of the trial or because they did not want to start catheter washouts. A few HCPs identified this as a potential challenge beyond the trial, as those without LTC complications might be reluctant to begin prophylactic washouts.

[some eligible patients] wouldn’t join the study because of it, because they didn’t want to run the risk of not being able to do the washouts. Jennifer, HCP3

We had a lot of the opposite and it’s quite interesting because we had a lot of people who were not on anything and absolutely didn’t want to be on anything. Keith, HCP2

### “… I do it myself … each week …” (fidelity of weekly washout behaviour and CATHETER II trial)

#### Participants’ adherence and commitment

Both trial participants and HCPs involved in the delivery of the trial shared similar views on the enactment of the trial and the washout behaviour. It was felt that most participants carried out their tasks for the entire duration of their participation without any barriers or complications. Participants often described the tasks, such as catheter washout, as a part of their routine. Participants also described how the calendar facilitated as a prompt or a reminder or both. While some participants required changes in the frequency of washout or washout type during the trial, there was no notable difference between how participants described their consistent commitment to the washout routine between groups (eg, arm of trial, gender or age). Only two participants (saline washout group) described being unable to washout successfully. All participants at the T2 interview stated that they would continue with their commitment until the end of the trial.

It’s [weekly washout] very much just part of a routine. Paul 71–80, ca

I’m quite happy to continue with this, I mean it is six months since I started it and it’s not an issue … if things really open and I go away for a week … I just take it with me. Rachel 69–70, sa

While a few participants in the standard care arm described being disappointed about the lack of opportunity for washout, most described completing the catheter calendar, the questionnaires and monthly phone call as per the trial protocol and described the benefits of the continued engagement in the trial.

it’s not a burden to me … I usually fill it in once a week anyway. Just when I change my catheter and my urine bag, and I just go through each week and carrying on the way that I am normally. Wendy 41–50, uc

I change the bag, I log it down on the calendar … they’re [calendar and phone calls] very good. Anderson 71–80, uc

Only two participants described not completing the calendar routinely due to a lack of interest or lack of time.

… he got to the stage where he wasn’t feeling very great and what have you … I think he was just getting tired and some days he just couldn’t be bothered to. Carer speaking on behalf of Mathew 91–100, uc

Most participants expressed being comfortable and positive about adhering to their randomised allocation and carrying out the weekly washout for the duration of the trial. They described that the commitments fitted well with their daily life. Some of them described their behavioural regulation and plans about how they would fit the washout routine with their routine catheter bag change on a particular day.

I can now do it [weekly washout] myself. My daughter did it for the first six months. But now she’s doing more hours at work, so I do it myself … I’m used to doing it now. Susan 51–60, ca

I do a washout every Saturday evening and change the catheter bag and I just report to her [at monthly phone call] anything that happens. Gorden 71–80, ca

Participants in the washout groups also referred to the positive outcome from the trial when describing their commitment. Many participants in the washout groups reported positive outcomes from their regular weekly washout. These included reduced blockage, pain, infection, reduced need for HCP support and greater psychological reassurance of reduced complications and their ability to self-manage potential complications.

It’s been amazing…my bladder spasms stopped … I used to bypass quite a bit and I’ve not bypassed, and I have not had any infections since we started doing the washouts at all. Kirsty 51–60, sa

Before I started this trial, I was having three or four blockages a year, right. I’ve only had one blockage in nearly a year. Ralph 71–80, ca

Some participants reported experiencing no impact of the weekly washout and one participant reported carrying out prophylactic washout prior to the trial. Two participants were unsure of the washout impact.

No impact as there was no problem. Steve 51–60, ca

Some who reported no impact of the washout felt the lack of change was due to the fact that they did not have any issues such as blockage and infection prior to the trial. Most of these participants were happy to continue with the washout routine regardless of its perceived lack of a positive outcome for them.

Understanding the definitive clinical impact of the trial on participants’ LTC-related complications was not possible within the scope of their role for all HCPs. Some, however, described that the participants had reported positive outcomes to them. HCPs reported that most participants were engaged with the trial and completed all necessary tasks, and adhered to the trial regardless of randomised allocation, including the standard care group who hoped to be in one of the washout groups.

I certainly have noticed a difference from the patients that are on the study as to the amount of blockages they’re getting and things like that. So you can see well hopefully this is a really positive thing. Jill, HCP6

Lot of people have had their letter to say that it’s come to an end and they’re quite disappointed because they do want answers. Ruth, HCP5

#### HCPs’ engagement

HCPs also described their continuous engagement in the trial as fitting within their professional role and responsibilities, finding it rewarding. They highlighted that the continuous support they received from the trial management team, encouragement from their colleagues, positive participant engagement and their personal belief in the importance of evidence-based practice contributed to the ease with which they could carry out their responsibilities.

I’m so glad we did because … patients really look forward to those monthly phone calls … and I have to say it’s a testament to the actual CATHETER II trial that it’s so well set up that I can leave as a research nurse and my colleagues can take over running the study with absolutely no hitch whatsoever to the patients and to the running of the trial. Keith, HCP2

I love doing these studies because you see a difference in what’s going on with the patients quite quickly. Jill, HCP6

HCPs reported that other than the challenges faced over the recruitment to the main trial, there were no barriers to trial delivery. They also described that the participants understood the trial well and most of them consistently enacted the tasks in their trial arm accordingly. They also did not report any notable differences between groups (eg, arm of trial, gender or age).

## Discussion

Through increased understanding of participants’ and HCPs’ views on and experiences of the CATHETER II trial, the findings provide a unique understanding of the barriers and enablers of behavior enactment, acceptability and satisfaction, and the potential implications for future policy and practice of LTC care. Both participants and HCPs highlighted that the trial was necessary, and the design and management of the trial were suitable and effective for both providers and recipients.

Consistent with the existing literature,^3 4^ we found that LTC-related complications are common, and these complications impact patients’ experience of LTC and their quality of life. These also constitute a significant burden on NHS resources, for example, the incidences of blockage and related complications require treatment by HCPs either at patients’ residences or at the hospital.

HCPs highlighted the current lack of guidance for prophylactic washout policies. This finding was not surprising given the limited evidence in the current literature on either the effectiveness of prophylactic catheter washout or the advantages and disadvantages of any type of washout solution over the other.^8^,^10^ They expressed a lack of confidence in the effectiveness of current practices in improving patient-centred outcomes. Therefore, robust evidence is crucial to inform understanding of the most effective practice to reduce LTC blockage and complications.

Our study shows that the CATHETER II trial design was acceptable, feasible and effective for both trial participants and HCPs. Participants in the washout group reported positive outcomes from the trial such as reduced blockage, pain or infection, reduced need for HCP support and greater psychological reassurance due to their ability to self-manage potential complications. This is consistent with the clinical analysis of the CATHETER II trial^12^ that reports favourable trends for lower rates of LTC blockages without a rise in S-CAUTI when employing prophylactic LTC washouts. Previous studies^25^,^27^ also suggest that enabling patients to effectively self-manage their illness could lead to reduced symptom severity, improved quality of life and lower healthcare costs.

Most participants in this study lacked washout knowledge or LTC skills prior to the trial, which underscores a significant clinical gap in existing patient care. The training provided in the trial was an essential element for the washout behaviour and was reported to be feasible and effective for participants receiving this and HCPs providing the training. When devolving routine care in patient self-management, provision of support is crucial so that individuals are confident and capable of managing their care appropriately. Our study found that catheter washout is an acceptable behaviour that could be enacted with fidelity given appropriate training.

Participants were willing and keen to self-manage their catheter and were able to conduct the washout behaviour without any additional support beyond their usual LTC care, after receiving training. Therefore, this study suggests that the washout training provided to LTC patients (as a part of the CATHETER II trial) led to effective patient self-management of LTC washout when needed. This training and education could empower patients in their self-management capabilities and confidence and help reduce the burden of LTC care on the NHS.^28^

Participants and HCPs expressed a thorough understanding of the HCPs’ role in the delivery of the trial and providing the washout training. Additionally, participants’ understanding and receipt of the training, and their ability to carry out the washout behaviour and complete calendar and phone calls were evident from both interviews and focus groups. This observation is consistent with existing literature that suggests that participants’ engagement with the intervention and use of intervention skills in daily life relates to treatment adherence and intervention effectiveness and that enactment of skills taught in the intervention relates to intervention effectiveness on behavioural outcomes.^29^,^31^

Unlike previous studies which showed a lack of evidence on effective strategies in participant retention and found diaries not to be helpful,^32^ this qualitative study suggested that the catheter calendar and monthly phone calls were a key component of the CATHETER II trial and might have contributed to participant retention and may also have important clinical translational considerations for optimising patient care and support. Although we did not directly assess retention strategies, participant retention was high between T1 and T2 within the qualitative study.

While a few HCPs indicated that patients without complications might be reluctant to undertake prophylactic washout beyond the trial context, most participants in this study appeared engaged regardless of their LTC experience. It is important to note that the findings from this study need to be considered in light of the small sample size of HCPs. Furthermore, as the patients in the study were already engaged in the CATHETER II trial, their views may not be representative of those that did not participate, as they were less inclined to engage in the washout phase.

### Conclusion

On a behavioural level, self-management of the weekly prophylactic washout, informed by a washout training toolkit and other aspects of the CATHETER II trial, was found to be acceptable, feasible and positively received by participants and HCPs. Participants in the washout groups reported positive outcomes. The catheter washout training and trial information were reported to be essential and suitable. Patients felt empowered in self-management of catheter care and integrated self-washouts into their routine without additional support as a result of the trial. The outcomes on acceptability, feasibility and effectiveness could inform catheter washout policies, potentially easing the burden on healthcare providers.

## supplementary material

10.1136/bmjopen-2024-087206 online supplemental file 1

10.1136/bmjopen-2024-087206 online supplemental file 2

## Data Availability

Data are available upon reasonable request.
