# Supplementary material for: Patient and healthcare professionals’ perception of weekly prophylactic catheter washout in adults living with long-term catheters: qualitative study of the CATHETER II trial
Source: BMJ Open. 2025 Apr 7;15(4):e087206. doi: 10.1136/bmjopen-2024-087206 (PMC11977465; doi:10.1136/bmjopen-2024-087206)
Supplement: online supplemental file 1 [file bmjopen-15-4-s001.docx]

Supplementary materials

**Supplementary table1: Topic guides**

T1 interview Topic Guide

T2 Interview Topic Guide

HCP focus group and interview Topic Guide

**Supplementary table 2: Coding Frame**

| **Theoretical Domains Framework (TDF)** | **Theoretical Framework of Acceptability (TFA)** |
| --- | --- |
| **Knowledge (of long-term catheter/blockage/ washout/scientific rational**  Related to the use of Catheter or washing it out (or lack thereof), Awareness of guidelines, or lack of awareness (available washout products, technique, times, frequencies)  Descriptions of research/evidence that would convince them to use a certain washout policy | **Intervention Coherence**  The extent to which the participants understood the trial process and catheter washout), burden (effort required to carrying the weekly washout and other aspects of the CATHETER II trial) |
| **Skills (needed for washout/catheter management/training)**  Skills development/Competence/Ability  Interpersonal skills/Practice Skill assessment, the training and relationship between health care professional and patient may facilitate weekly catheter washout | **Self-efficacy (including that of the carer if applicable)**  The participant's confidence that they can perform the behaviour(s) required to participate in the intervention. How confident participants were/believed to be to carry out the weekly washout out and other elements of the trial |
| **Beliefs about consequences (self, carer or for research)**  Outcome expectancies/Characteristics of outcome  Expectancies/Anticipated regret/Consequents  *Positive and negative outcomes from taking part in the study. Beliefs about treatment outcomes – both theoretical and based on experience due to using weekly washout. Descriptions/explanations of if a particular washout is effective. Potential long-term outcomes of using weekly washout*  *(anticipated regret or negative consequence also coded under opportunity cost)* | **Perceived effectiveness (**could be optimism)  The extent to which the weekly catheter washout or their taking part in the trial will be/was beneficial and is perceived to (or have) achieve its purpose |
| **Optimism**  Optimism/Pessimism/Unrealistic optimism  about the outcome from the weekly catheter washout or the trial, belief about whether washout will or will not give them a positive outcome, hypothetical – optimistic about the outcome of the study  *(Pessimism also coded under burden)* | **Affective attitude**  Participants’ feeling about catheter washout and taking part in the trial prior to /After taking part  *(Code understanding of the intervention component under intervention coherence)*  *(Code feelings about effectiveness under perceived effectiveness)* |
| **Beliefs about capabilities** (washout/trial related commitment. Including capabilities to receive support or capabilities of the carer)  Self-confidence/Perceived competence/Self-efficacy  Perceived behavioural control/Beliefs/Self-esteem  for catheter washout and/or trial related commitment, how easy or difficult it will/would be to do the weekly washout or catheter care related tasks, how confident a participant feels that they would be able to follow the washout training | **Burden**  The perceived (and actual) amount of effort required to carrying the weekly washout and other aspects of the CATHETER II trial  (to be combined with opportunity cost for T1 interviews) |
| **Professional/social role and identity** (self/HCPs)  Professional identity/Professional role  Social identity/Professional boundaries/Professional confidence/Group identity/Leadership/Organisational commitment in carrying out weekly washout and other trial tasks in certain way because of their personality, or personal commitment or identity | **Opportunity cost**  The extent to which benefits, profits, or values must be given up to engage in the CATHETER II trial.  (to be combined with burden for T1 interviews) |
| **Environmental context and resources** (Physical resources)  Environmental stressors/Resources/material resources/Organisational culture/climate  Salient events/critical incidents/Person/environment interaction/Barriers and facilitators related to allocated washout, catheter management or other trial commitments, training *prior catheter treatment and resources, advice and services received, HCPs support, whether or not a certain catheter care pathways were prescribed* | **Ethicality**  The extent to which CATHETER II trial had good fit with an with the participants’ personal values |
| **Memory attention and decision process (T1 prospective)**  *Memory/Attention/Attention control*  *Decision making/Cognitive overload/tiredness*  for managing catheter washout, monthly phone call and catheter calendar. At T1 code participant’s descriptions of when they think they would forget as well as reasons why they don’t think they would forget at this domain and description of any strategies |  |
| **Intentions**  Stability of intentions/ A conscious decision to perform a behaviour (related to allocated washout, catheter management or other trial commitment) in a certain way, how inclined they are carryout weekly washout, inclinations to be complete the study, when they are and are not inclined to not do the weekly washout.  (i.e. ‘Beliefs about Consequences’)  Note: Indicator of intention must be explicit and not inferred |  |
| **Motivation and Goals**  Goals (distal/proximal)/Goal priority/Goal/target setting/Goals (autonomous/controlled)/Action planning Implementation intention of any plan or target behaviour or outcome. Whether  weekly wash out is a priority. How carrying out weekly washout is (or is not) in conflict with other daily life routines  or responsibility (goal conflict)  (including desire to contribute to research) |  |
| **Behavioural regulation (T1 prospective)**  aimed at managing or changing objectively observed or measured actions managing catheter washout and catheter calendar. Self-regulatory strategies already in place that would influence the weekly washout.  Coping plans, problem solving. Strategies used in response to any potential hurdles form health care professionals or resources.  **(**Strategies to remember to do the weekly washout code at ‘Memory’) |  |
| **Social influences (**including but not limited to that of carer)  *Social pressure/Social norms/Group conformity/Social comparisons/Group norms/Social support/Power, Intergroup conflict/Alienation, Group identity*  *Modelling* about how others influence whether or not a particular washout policy is adopted including influence of other healthcare professionals, family members/friends support or lack thereof, others attitude and any need of their support (instrumental or emotional) |  |
| **Emotion (around catheter management/washout)**  Fear/Anxiety/Stress/Depression  Positive/negative affect/ Burn-out *experienced by participants while carrying out weekly washout, about doing the specific washout. Include ‘no’ answers*  *(Descriptions of HCPs’ emotions regarding the participants washout code at Environmental resources, friends or families’ emotions code at ‘Social influence’)* |  |
| **Reinforcement (**Reinforcement/reward of catheter washout based on past experiences that is not knowledge**)**  Incentives/Punishment/Consequents/Reinforcement  Contingencies/Sanctions  *Also code ‘no’ answers* |  |
| **Nature of behaviour:**  Catheter Blockage or any LTC experience relevant to washout (coded here in addition to LTC experience) |  |
| **Inductive codes:** |  |
| **LTC Experience** (emotion/past experiences that is not knowledge) Catheter related AE/ Catheter Non AE |  |
| **Awareness (or experience) of washout prior to trial** |  |

**Supplementary table 3: Themed quotes table**

| **Themes** | **Quotes** |
| --- | --- |
| **1 Acceptability of CATHETER II trial and weekly washout** | |
| Positive affective attitude and perceived effectiveness | *I’d never heard that you could flush them out. I just thought that was them and they were changed every twelve weeks and you got a new one, and that was it. Steve 51-60, ca*  *I don’t really know what types (washout) they are … until I know that they’ve tried these both types I’m not going to find out am I. David 71-80, ca*  *I’ve had a number of urinary infections and I’ve also had a blockage in the catheter at one stage, if we can stop any of those things then that’s great. Philip 71-80, sa*  *with having things like clots or sediment and things, it would be nice to see if that did reduce the amount of that. Elizabeth 20-30, sa* |
| Perceived lack of negative consequences, opportunity cost or burden | *I think my decision was really based on the fact that, I mean, what have I got to lose? … this is like a potential new thing for me to try, so I thought why not give it a go. Melissa 20-30, ca*  *I mean it can’t do any harm, and if it does any good then I’m on a winner. Kenny 71-80, uc* |
| Contribution to knowledge | *I think there’s got to be more education when they fit a catheter of what’s going to happen, that’s the bit that in the future that other people would benefit. Bruce 81-90, ca*  *Well I suppose for myself it all depends which one I have, but the end of it is, is a case of helping other people, if it helps other people to stop their catheter being blocked then I’m all for it. Verginia 71-80, sa* |
| HCPs: evidence need for policy and practice | *The district nurses were quite happy for us to come on board with this but we had to obviously clear it because the policy now is no washouts at all. Wendy, HCP4*  *We don’t know whether what we’re doing is good or bad or whether it’s useful at all beyond the anecdote. Keith, HCP2*  *At the moment, there is no recommendation that there should be any prophylactic washouts using any solutions and that’s not a reflection on an evidence but it is a reflection on the fact of the lack of evidence, so everything is balance of benefits and risks. If you do washouts, then you are trying to reduce the risks of blockages and therefore risks of bypass and the blockages can cause a retention, and obviously can cause increased use of, well, pain for the patient first and could then emergency that they’re having a retention, having to get the nurse out or go themselves to Accident & Emergency, which is obviously not a good thing for to be happening frequently for anyone. But, on the other hand, if you’re doing regular prophylactic washouts or doing prophylactic washouts carries the risk of introducing an infection or making an infection a little bit worse, therefore… hence the catheter trial was there to say, “Okay, we will look if there is an evidence that if having prophylactic, regular prophylactic washouts would actually improve blockage or not, and will it increase the risk of infection or not. Lawrence, HCP7*  *I think it’d just be nice to know if they’re beneficial at all because … sometimes you feel like you’re going in and doing something that maybe isn’t having an impact on the outcome for the patient. So yeah, it’d be good to see if evidence shows either way, are they actually useful or not. Susan, HCP 1*  *In our health board they had stopped doing washouts and they said it was policy now that if a catheter was blocked you didn’t attempt a washout, you just changed the catheter… well eight years ago, we never would have done that, we always tried a washout because quite often a washout would work, so I was quite baffled that they’d changed that. Wendy HCP4*  *We used to always try and do the PH of the urine… if it was above seven we’d go for a citric acid washout, if it was below seven we might try normal saline to start with ... I think now we kind of rely on the PH less and we go by the symptoms or what the catheter’s blocking with… it’d be interesting to see what CATHETER II brings out with that. Susan, HCP1* |
| **2. Feasibility of CATHETER II trial and weekly washout** | |
| Perceived ability | *Once I’ve been shown how to do I think I can do it. I mean I was shown how to do insulin injections, now I do it myself. I think if somebody shows me I can do it myself. Bruce 81-90, ca*  *What I might do is do it when my support worker is around so that she can see what I do and if there are any problems, she can help. Lorraine 61-70, uc*  *I would probably have to go up to the hospital to get a demonstration on how the application – if I was picked for the saline or the weak acid, they would probably show me how to do it when I change my leg bag. That would probably be the best time when something like that would be done. As far as I understand it’s all done by gravity anyway […] Once I get the training, that would be all right. Richard 71-80, ca*  *once he’s had the training and he knows where he’s going he’ll be fine. Career speaking on behalf of Mark 71-80, ca*  *From the information I’ve gleaned from the information pack we received that it will be a very straightforward procedure. As I say, with one session of training, I can’t see any real problems. Stella 71-80, sa*  *My husband does the flush outs for me. We do it once a week on a Monday. But it’s been no problem. Painless because my husband does it and I don’t have to wait for the district nurses to come out. Ruth 61-70, ca*  *it was good, it was quite straightforward once it’s sort of explained to you and as I say, it takes a little bit of getting used to but once you’ve done it a few times then it’s more easy to do. Stephanie 41-50, sa*  *My wife’s here and she will help if I need help but I’m quite able to do it. Philip 71-80, sa* |
| Intervention coherence, self- efficacy and resources needed | *He got to the stage where he wasn’t feeling very great and what have you…I think he was just getting tired and some days he just couldn’t be bothered to. carer responding on behalf of Mathew 91-100, uc*  *that was very simple because all she was very good. She showed me what I... well, she guided me... like, I done it myself, but she guided me along to what I had to do, it was really simple and she said, “Well, can you manage in future?” I said, “Yes, that would be fine.” It’s very simple. You know, there’s not really a lot to it… You I haven’t had any problems doing it. Virginia 71-80, sa*  *there was a video online […] All looked very straightforward, and I followed the instructions and had no problems. Kevin 71-80, sa*  *it was designed in a way that it would be self-care with the washouts because that means that you do not have to add a lot of workload for the already stretched district nurses, and if it wasn’t this way, it would have been a big obstacle in implementing the change of prophylactic washouts if they had to be delivered by the nurse or the healthcare assistant. Lawrence, HCP7*  *It was easy, the patients followed the instructions. They were able to look at it before we actually come online to them as well to go through it, so they’d gone through the video prior to us coming on and watching them do the procedure themselves, so yeah, it was good. Jill, HCP6*  *a lot of the ones that were done remotely they went really well and the participants were really happy with that, but ..We found that some of the participants were okay with IT and some were not so good. Ruth, HCP5*  *I definitely see that if somebody is having no issues [with LTC], they would be reluctant to rock the boat or sort a problem that does not exist. Lawrence, HCP7* |
| **3. Fidelity of the CATHETER II trial** | |
| Participants’ adherence and commitment | *Every Thursday I do my washout*. *Elizabeth 20-0, sa*  *It was quite interesting as well from a research point of view, the number of patients that are joining the study and everyone has done what they’ve been randomised. Jill, HCP6* |
| HCP engagement | *I love doing these studies because actually you see a difference in what’s going on with the patients quite quickly. Jill, HCP6*  *the trial managers have been very well on-hand. They have implemented the video induction which was quite helpful because it meant that the nurses can have their induction visit even more than once if we required but also we can have it separately…the trial managers have been on-hand for troubleshooting at any time… the units felt quite well supported by this. The fact that most of the questions were sent centrally it meant that less burden on the centres, but obviously there were the requirement to have the monthly phone call, and the monthly phone call was the important part because it kept these patients, or participants, engaged with the study; they felt that there is somebody that is there to, not necessarily look after them, but to make sure that they can talk to someone about it. Lawrence, HCP7*  *I went and talked to my boss and said, “Look, could we have a look at this?” and they said, “Yeah, this would be great” … it was my passion that pushed it to our research department, and I’m so glad we did because … patients really look forward to those monthly phone calls … they really valued my contact, and I have to say it’s a testament to the actual CATHETER II trial that it’s so well set up that I can leave as a research nurse and my colleagues can take over running the study with absolutely no hitch whatsoever to the patients and to the running of the trial. Keith, HCP2*  *it’s a shame that we didn’t recruit as many as we should have because from our point of view COVID affected that greatly. Wendy, HCP4*  *I think our major barrier that we had was actually getting a hold of the list off the bowel and bladder service. It took us months to get that but as soon as we got that list, we recruited the number of patients we could recruit because we sent a letter out to every single person on that list who was still alive or not in a nursing home, and basically the recruitment figure we got was everybody who said yes after sending everybody a letter. Keith, HCP2* |
