# Supplementary material for: Patient and healthcare professionals’ perception of weekly prophylactic catheter washout in adults living with long-term catheters: qualitative study of the CATHETER II trial
Source: BMJ Open. 2025 Apr 7;15(4):e087206. doi: 10.1136/bmjopen-2024-087206 (PMC11977465; doi:10.1136/bmjopen-2024-087206)
Supplement: Supplementary file 3 [file bmjopen-2024-087206.reviewer_comments.pdf]

PEER REVIEW HISTORY

BMJ Open publishes all reviews undertaken for accepted manuscripts. Reviewers are asked to complete a checklist review form and are provided with free text boxes to elaborate on their assessment. These free text comments are reproduced below.

ARTICLE DETAILS

Title (Provisional)

Patients and health care professionals' perception of weekly prophylactic catheter washout in adults living with long-term catheters: Qualitative Study of the CATHETER II Trial

Authors

Tripathee, Dr Sheela; Abdel-fattah, Mohamed; Johnson, Diana; Constable, Lynda; Cotton, Seonaidh; Cooper, David; MacLennan, Graeme; Evans, Suzanne; Young, Amanda; Dimitropoulos, Konstantinos; Hashim, Hashim; Kilonzo, Mary; Larcombe, James Hugh; Little, Paul; Murchie, Peter; Myint, Phyo Kyaw; NDow, James; Paterson, Catherine; Powell, Karen; Scotland, Graham; Thiruchelvam, Nikesh; Norrie, John; Omar, Muhammad Imran; Maclennan, Sara J

VERSION 1 - REVIEW

**Reviewer**	**1**
**Name**	**Mackay, William**
**Affiliation**	**University of the West of Scotland, Biological Sciences and Health**
**Date**	**03-Jun-2024**
**COI**	**None**

Dear colleagues, thank you for the opportunity to review your manuscript.

In the methods section you say "To enrich rigour, selected transcripts and thematic outputs emerging from the interviews were

read by multiple researchers,...". Can you be specific? How were they selected? How many researchers were involved? Was there consistency across all selected manuscripts?

In the results section you say "Six HCPs participated in a focus group and one HCP was interviewed." With one interview - was there a risk of bias in the response? Single experience? I appreciate that it is difficult to organise interviews with busy HCWs.

Result section presented clearly. Conclusions backed up by results.

Best of luck.

**Reviewer**	**2**
**Name**	**Choudhary, Sidhya**
**Affiliation**	**Government Medical College and Hospital**
**Date**	**27-Jun-2024**
**COI**	**None**

The article needs a second look at English grammar. Abbreviations should be in square brackets and placed before a full stop. Ensure all references are formatted consistently and correctly according to the journal's guidelines.

Abstract (Page 4):

- **Line 27-28**: Clarify the objectives for healthcare professionals (HCPs) or the objectives of researchers. Correct the statement.
- **Line 34 and 42**: These should be under a common heading of methodology. Specify whether this was a single-centre or multi-centric study.

Introduction (Page 7):

- **Line 12**: Specify what various complications are referred to.
- **Line 35**: Integrate the mention of specific study publications more seamlessly. For instance, instead of stating "The details of the CATHETER II trial and outcomes are reported in 2 separate publications," briefly summarize the key findings or importance of those publications to provide context for the qualitative study.
- **Overall**: Although the introduction is concise, providing a brief overview of the current evidence on LTC washouts (saline vs. citric acid) would enhance the reader's understanding of the study's background.

Methods Section (Page 8-10):

- (a) The section could benefit from better organization and clearer flow.
- (b) Ensure consistent formatting throughout the section, including the alignment and spacing of text. This will improve readability.
- (c) The recruitment process description is somewhat fragmented. Consolidate information about participant consent, sampling strategy, and contact methods into a coherent paragraph. Provide more details about the participants, such as demographics and the rationale for selecting the specific sample size.
- (d) Specify the exact dates for the data collection period, i.e., "from February 2020 to August 2023," to provide clear context.
- (e) While the use of TDF and TFA is mentioned, providing a brief explanation of these frameworks within the methodology section itself would enhance understanding for readers unfamiliar with them.
- (f) Indicate how many patients gave interviews by themselves and in how many cases data were retrieved from caregivers.
- (g) Define how a candidate for washout was determined.

Table 1 (Page 11):

- No point in using pseudonyms. Were there any patients in which both saline and citric acid were used? Clarify how it was decided whether a patient would receive a saline or citric acid wash.

Results Section (Page 12-21):

- (a) Remove Patient Dialogues: Eliminate all patient dialogues from the manuscript. These are not suitable for publication and detract from the scientific value of the paper. Replace these dialogues with succinct descriptions of patient symptoms, treatment responses, and outcomes, focusing on the data that supports their conclusions.
- (b) Rewrite Clinical Descriptions: Replace the dialogues with concise clinical descriptions that highlight the patient's symptoms, treatment, and response. Focus on the clinical and microbiological findings that are central to the case.
- (c) Improve Structure and Flow: Restructure the manuscript to follow a logical progression from patient presentation through diagnosis and treatment to outcomes. Ensure each section transitions smoothly to the next.
- (e) Adhere to Academic Standards: Maintain a formal, objective tone throughout the manuscript. Avoid subjective or anecdotal elements that do not contribute to the scientific discussion.

Discussion Section (Page 19-21):

- (a) Reorganize the Structure: Restructure the discussion to follow a clear, logical progression. Start with a summary of key findings, discuss their implications, and conclude with recommendations for future research and practice. Ideas are presented without a clear, logical flow, making it difficult to follow the main points. Example: The mention of existing literature (lines 23-24 and 37-41) is scattered throughout the text without a cohesive connection to the main findings.
- (b) Condense and Clarify: Condense the text to make it more concise and focused. Avoid repetition and ensure each paragraph contributes to the overall narrative. Example: P 20, L 25- Participants in the washout group reported positive outcomes from the trial such as reduced blockage, pain or infection, reduced need for HCP support and greater psychological reassurance due to their ability to self-manage potential complications.; AND P 20, L40 "Our study found that catheter washout is an acceptable behaviour that could be enacted with fidelity given appropriate training."
- (c) Focus on Key Points: Highlight the most important aspects of the trial, such as the effectiveness of washout training, patient self-management, and the need for clear guidelines on prophylactic catheter washout

---

**Reviewer**	**3**
**Name**	**Alex, Joby**
**Affiliation**	**Western sydney Local Health district**
**Date**	**18-Dec-2024**
**COI**	**nil**

---

1. What is a ‘longitudinal’ qualitative study – is this more a process evaluation?

2. Sample size justification n=50, why were 50 selector?

3. 7 HCPs participated, why are the number of HCPs and patients not the same – justification of the n=7 sample size is required.

4. Conclusion explains that 'acceptability', and 'feasibility' were demonstrated – although these constructs were not measured. This seems an inappropriate conclusion as qualitative research explores these issues and doesn't establish this, as such. – this needs revision.

5. There needs to be a better sample size justification.

6. How were the HCPs selected and sampled to be involved?

7. RESULTS – the quotes selected are very short in nature. This perhaps reflects the surface and superficial nature of the data collection. More in-depth and rich quotes would be preferred.

8. How was data saturation known to be achieved?

9. Thanks for including your interview guide. The question route is very exploratory around the trial, however, there appears to be very limited questioning on translation to routine care, understanding of implementation factors and barriers, facilitators, and sustainability in routine practice. (e.g economic impact to the health system and patients, for example). The question guide seems very surface in nature.

10. Caution with terminology such as 'demonstrated' in qualitative research – See ") this qualitative study demonstrated that the catheter calendar and monthly phone calls were a key component of the CATHETER II trial and might have contributed to participant retention and may also have important clinical translational considerations for optimising patient care and support" – the aim of qualitative research is primarily to explore, and not demonstrate.

11. There is no 'Limitations' section – the study limitations are not acknowledged. Bladder washouts may need to be considered carefully or contraindicated for those who are at risk of developing autonomic dysreflexia, or patients with neurogenic bladder conditions. Other aspects such as strategies for minimising the risk of infection while flushing the catheter, precautions for those who experience bladder spasms, and small compliance bladder may need to be explored further.

Introducing the catheter flushes as a prophylactic measure may not eliminate the root cause of the blockage. Consider exploring the contributing factors of catheter blockages and how to address them.

VERSION 1 - AUTHOR RESPONSE

**Reviewer 1**	
In the methods section you say "To enrich rigour, selected transcripts and thematic outputs emerging from the interviews were read by multiple	***We have amended the text and have mentioned this information. Manuscripts of the transcripts were read by two researchers. In addition, we have also mentioned the number***

researchers,...". Can you be specific? How were they selected? How many researchers were involved? Was there consistency across all selected manuscripts?	***to transcripts and information about the consistency in data have now been added in the Method section.***
In the results section you say "Six HCPs participated in a focus group and one HCP was interviewed." With one interview - was there a risk of bias in the response? Single experience? I appreciate that it is difficult to organise interviews with busy HCWs.	***We recognise that the number of HCP participants is lower than we anticipated due to the difficulty in recruiting HCPs. However, the views are representative of six out of the 21 recruiting sites., There was a similarity in views from the focus group and the in-depth interview that was conducted following the focus group, and the research team considered the data being adequate. We, however, have stated this as a limitation to be considered.***
Result section presented clearly. Conclusions backed up by results.	**Thank you for your feedback. No changes required.**
**Reviewer 2**	
The article needs a second look at English grammar. Abbreviations should be in square brackets and placed before a full stop. Ensure all references are formatted consistently and correctly according to the journal's guidelines.	***Thanks for pointing this. The manuscript has been read by native English Speaker. Grammatical errors are now corrected throughout the manuscript. The abbreviations and reference brackets are now changed and formatted in line with BMJ Open guidelines.***
**Abstract (Page 4):**<br>**Line 27-28:** Clarify the objectives for healthcare professionals (HCPs) or the objectives of researchers. Correct the statement.	***This has now been corrected by stating that "*****The objectives of the healthcare professionals (HCPs) focus group and interviews were to explore their attitudes towards weekly prophylactic catheter washout and views on the provision of training, and participants' ability to enact washout behaviours"**
**Line 34 and 42:** These should be under a common heading of methodology. Specify whether this was a single-centre or multi-centric study.	***This has now been clarified by stating that 'the participants were recruited from multiple trial sites.***
**Introduction (Page 7):**<br>**Line 12:** Specify what various complications are referred to.	**Specific complications are now added in the Introduction section stating. We have added the following: "*****various complications, including LTC blockage, pain, bladder spasm, symptomatic catheter-associated urinary tract infection (S-CAUTI) and urinary leakage.***
**Line 35:** Integrate the mention of specific study publications more seamlessly. For instance, instead of stating "The details of the CATHETER II trial and outcomes are reported in 2 separate publications," briefly summarize the key findings or importance of those publications to provide context for the qualitative study.	***Brief summary of outcome from the CATHETER II clinical paper is now added in Introduction section page 6.***
**Overall:** Although the introduction is concise, providing a brief overview of the **current evidence on LTC washouts (saline vs. citric acid) would enhance the reader's understanding of the study's background.**	***Further detail on the current evidence and guidelines on the washouts is now added in the Introduction section to state that "*****prophylactic washouts is not recommended in current guidelines to prevent blockages. Evidence on benefits or potential harms associated with prophylactic washout practice with either saline or acidic liquids to prevent blockages and other LTC-related adverse events compared to no washout policy is reported to be of low quality and insufficient.**

**Methods Section (Page 8-10):**<br>(a) The section could benefit from **better organization and clearer flow.**	***The Methods section has now been reorganized to improve flow as suggested.***
(b) Ensure consistent formatting throughout the section, including the alignment and spacing of text. This will improve readability.	***Section has now been formatted consistently throughout.***
c) The recruitment process description is somewhat fragmented. Consolidate information about participant consent, sampling strategy, and contact methods into a coherent paragraph.<br><br>Provide more details about the participants, such as demographics and the rationale for selecting the specific sample size.	***The section has been edited accordingly to present the recruitment process logically.***<br><br>***Participant demographics are presented in result section.***<br><br>**Sample size justification is now presented in Methods: Recruitment section to clarify that we used the principles of 'information power' for planning for adequate sample size. Information power indicates that number of participants needed is based on the information the sample holds. It is suggested that the size of a sample with sufficient information power depends on the aim of the study, specificity of the sample, use of established theory, quality of conversation, and analysis strategy (19).**<br><br>**Our study had relatively narrow aim and focus; dense sample within each arm of the trial and associated overall sampling characteristics of purposive sampling; interviews to be conducted with same participants at two time points, analysis to be guided by well-established theories such as theoretical domains framework and theoretical framework of acceptability; and strong research team with expertise and extensive experience qualitative research and similar studies ensuring the dialogue is anticipated to be high quality.**<br><br>**Considering these points, we anticipated that total of 40 participants proportionate in three arms of the trial as an adequate sample size to meet our objectives.** ***Due to the loss of participants for the follow up (T2) interviews additional 10 participants were recruited making number of participants 50.***<br><br>**Focus groups with Health care professional involved in the trial was designed to supplement the findings from patient interview data. Given the small number of HCPs involved in the trial, focus group data from 1st group (including 6 HCPs) from six different trial sites was further checked by conducting interview with additional HCP. Data from focus group and the following interview were consistent. Therefore, data from HCPs was considered adequate.**
d) Specify the exact dates for the data collection period, i.e., "from February 2020 to August 2023," to provide clear context.	***This has been edited accordingly to state that data was collected "from February 2020 to August 2023,"***
e) While the use of TDF and TFA is mentioned, providing a brief explanation of these frameworks within the methodology section itself would enhance understanding for readers unfamiliar with them.	**Further detail on the frameworks has now been added in method: Data analysis section to state that**<br>***The TDF is an integrated theoretical framework that guides content analysis of qualitative data within defined domains of behaviour change such as knowledge, skills, self-confidence, optimism, intentions, goals, memory and attention, behavioural regulations, and environmental and social context. It helps to rigorously identify the behavioural***

	processes or barriers and facilitators involved in behaviour change and assess implementation processes. (19-22) TFA provides a comprehensive guide for the assessment of acceptability of healthcare interventions and is used to inform a robust understanding of acceptability across core constructs such as Intervention coherence, self-efficacy, perceived effectiveness, attitude and burden.
(f) Indicate how many patients gave interviews by themselves and in how many cases data were retrieved from caregivers.	***Details on patients interviews by self or caregivers has now been added to state that 3 participants had their caregiver answering the question on their behalf while the participants were also present in the room.***
(g) Define how a candidate for washout was determined.	***This has been clarified in methods section by stating that Patients were randomly allocated to one of the three trial arms (saline, acid washout or usual care group).***
**Table 1 (Page 11):**<br>No point in using pseudonyms. Were there any patients in which both saline and citric acid were used? Clarify how it was decided whether a patient would receive a saline or citric acid wash.	***Pseudonyms are used in line with the BMJ Open guidelines for anonymization of transcript and excluding any direct identifier.***<br><br>***Patients did not use both washout at the same time, but 1 patient moved from citric acid to saline washout and 1 patient moved from saline washout to usual care and this has been stated in the Table 2.***<br><br>***Patients were randomly allocated to one of the three arms of the trial (saline, acid washout or usual care group). This has been clarified in the Methods section.***
**Results Section (Page 12-21):**<br>(a) Remove Patient Dialogues: Eliminate all patient dialogues from the manuscript. These are not suitable for publication and detract from the scientific value of the paper. Replace these dialogues with succinct descriptions of patient symptoms, treatment responses, and outcomes, focusing on the data that supports their conclusions.	***Thanks for your advice. The authors believe that in reporting results in qualitative research representative quotations from the participants are important to rigorously illustrate the findings and to demonstrate how the findings and interpretations have arisen and to ensuring that findings are grounded in the data. This is a standard practice in qualitative research that has been followed*** *(References: Merriam, S. B., & Tisdell, E. J. (2016). Qualitative Research: A Guide to Design and Implementation (4th ed.). San Francisco, CA: Jossey-Bass.*<br>*Corden, A., & Sainsbury, R. (2006). Exploring 'Quality': Research Participants' Perspectives on Verbatim Quotations. International Journal of Social Research Methodology: Theory & Practice, 9(2), 97–110*<br>*Braun V, Clarke V Using thematic analysis in qualitative research. Qualitative Research in Psychology 3 (2), 77-101.*<br><br>***Use of quotations in this paper also confirm to a widely accepted (and in line with BMJ) guidelines for reporting qualitative research (Consolidated criteria for Reporting Qualitative research) COREQ (Tong et al., 2007) which highlights the effective application of quotations.***
(b) Rewrite Clinical Descriptions: Replace the dialogues with concise clinical descriptions that highlight the patient's symptoms, treatment, and response. Focus on the clinical and microbiological findings that are central to the case.	***The clinical findings from the CATHETER II has been published in BMJ Open as a separate publication (Reference 12- Abdel-Fattah et al., 2024).***<br><br>***The aim of this embedded qualitative study was to explore trial participants' experience of long-term catheters (LTC), the acceptability of washout policies, their experience of the CATHETER II trial and their satisfaction with outcomes from the CATHETER II trial, and HCPs attitudes towards weekly prophylactic catheter washout and views on the provision of training, and participants' ability to enact wash-out behaviours within the CATHETER II trial. Therefore, the results***

	are presented accordingly to reflect analysis of patients and health care professionals' perspectives on feasibility, acceptability and fidelity of CATHETER II trial and catheter washout on a behavioural level. Clinical Descriptions of the participants is outside the scope of this qualitative study.
(c) Improve Structure and Flow: Restructure the manuscript to follow a logical progression from patient presentation through diagnosis and treatment to outcomes. Ensure each section transitions smoothly to the next.	***As stated in the above response this was an embedded qualitative study that was designed to explore CATHETER II trial participants' experience, the acceptability of washout options, their experience of the CATHETER II trial and their satisfaction with their trial outcomes. The objectives of the healthcare professionals (HCPs) focus group and interview were to explore their attitudes towards weekly prophylactic catheter washout and views on the provision of training, and participants' ability to enact wash-out behaviours within the CATHETER II trial. Therefore, Results section are presented according to key findings around acceptability, feasibility and fidelity of the catheter II trial based on treatment and outcome. Presenting patient presentation and diagnosis is outside the scope of this qualitative study and are presented in a separate publication (Reference 12- Abdel-Fattah et al., 2024)***
**(there was no bullet point 'd')**<br>(e) Adhere to Academic Standards: Maintain a formal, objective tone throughout the manuscript. Avoid subjective or anecdotal elements that do not contribute to the scientific discussion.	***The use of language has been reviewed throughout the manuscript and edited as appropriate. However, we believe that the subjective narratives presented align well with study design and objectives of this qualitative study, as this embedded qualitative study was designed with the post-positivist/interpretivist) epistemological approach (Guba & Lincoln, 1994) that social reality can be subjectively interpreted through exploration of people's understandings of their world. Therefore, a focus on understanding the individual context through the interpretations of participants help understand their attitude, experience and behaviours.***
**Discussion Section (Page 19-21):**<br>(a) Reorganize the Structure: Restructure the discussion to follow a clear, logical progression. **Start with a summary of key findings, discuss their implications, and conclude with recommendations for future research and practice**. Ideas are presented without a clear, logical flow, making it difficult to follow the main points. Example**: The mention of existing literature (lines 23-24 and 37-41) is scattered throughout the text without a cohesive connection to the main findings.**	**The Discussion section is revised to improve the flow. The mention of the existing literature relates to strength of the trial design that could inform future research with regards to fidelity of trial.**
(b) Condense and Clarify: Condense the text to make it more concise and focused. Avoid repetition and ensure each paragraph contributes to the overall narrative. Example: P 20, L 25- Participants in the washout group reported positive outcomes from the trial such as reduced blockage, pain or infection, reduced need for HCP support and greater psychological reassurance due to their ability to self-manage potential complications.; AND P 20, L40	**We have reviewed the Discussion section and made some changes.**<br><br>**Both statements in the example are considered necessary by the authors and not a repetition because the page 20 line 25 discusses the outcome from weekly prophylactic washout whereas p20 line 40 reports the acceptability of and fidelity of the weekly prophylactic washout.**

"Our study found that catheter washout is an acceptable behaviour that could be enacted with fidelity given appropriate training."	
(c) Focus on Key Points: Highlight the most important aspects of the trial, such as the effectiveness of washout training, patient self-management, and the need for clear guidelines on prophylactic catheter washout	**The Discussion section is now reformatted to highlight as the effectiveness of washout training, patient self-management, and the need for clear guidelines on prophylactic catheter washout.**
**Reviewer: 3**	
**1. What is a 'longitudinal' qualitative study –** is this more a process evaluation?	***This study embedded in the CATHETER II trial collected repeated data (once before the trial and during the trial) to explore participant experience both before and after the trial. While this study explored the effectiveness of various element of both the trial and the intervention it was not primarily the process evaluation of the trial.***
2. **Sample size justification** n=50, why were 50 selector?	**Justification for the sample size is now added in the methods: recruitment section to clarify that *Sample size was planned by following the principles of Information power (19). Given relatively narrow aim and focus; dense sample within each arm of the trial; interviews to be conducted with same participants at two time points, analysis to be guided by established theories; and strong research team with expertise ensuring high quality conversation with participants and additional data from HCPs, sample size of 40 patients proportionate in three arms of the trial was considered an adequate and sufficient to provide descriptive and interpretive depth needed to clearly describe theoretical underpinnings and meet study objectives. As explained in the method section a due to the loss of participants for the follow up (T2) interviews additional 10 participants were recruited making the total number of participants 50.***
3. 7 HCPs participated, **why are the number of HCPs and patients not the same –** justification of the n=7 sample size is required.	***This study was not comparing the perspectives of Patients and HCPS on a same issue but rather exploring their views and experience on matters relevant to them or their separate role/ circumstances there is not a need for the sample size to be the same both groups.***<br><br>***Considering all elements that could impact the information power and experience from previous similar studies the team decided to conduct interviews with 40 patients (as described in the sample size justification response above) from the cohort of potential 600 CATHETER II trial participants.***<br><br>***Focus group were conducted with health care professional involved in the trial from across 21 sites. The cohort of the health care professionals was much small. 1 focus group with 6 HCPs (from 6 of the 21 trial sites) was conducted. After preliminary analysis of the focus group data, one further interview was conducted to check the data from the focus group and the team felt that no new information was arising. Therefore, focus group data was considered adequate. (This has now been added to Methods section)***
4. Conclusion explains **that 'acceptability', and 'feasibility' were demonstrated – although these constructs were not measured**. This	**We have now rephrased the term demonstrated with more suitable terms such as 'expressed' or 'suggested'. However, we believe that the exploration of both patients and health care professionals' perspectives in this study and analysis**

seems an inappropriate conclusion as qualitative research explores these issues and doesn't establish this, as such. – this needs revision.	**guided by theoretical underpinning from TDF and TFA provides the evidence on acceptability and feasibility of the self-management of weekly prophylactic washout and the CATHETER II trial on a behavioural level, as presented in result section *'…I like the idea of washout…' (acceptability of weekly washout behaviour and CATHETER II trial)* and section *"…it's quite straightforward once it's sort of explain to you…' (feasibility of weekly washout behaviour and CATHETER II trial)* in this study.**
5. **There needs to be a better sample size justification.**	***As described in previous responses to (Reviewer 2 comment Methods section c, Reviewer 3 comment 2 and 3) Sample size justification has now been added to the Methods section.***
6. **How were the HCPs selected and sampled** to be involved?	**All HCPs involved in the CATHETER II trial were invited to take part in focus groups or for interview and all interested HCPs were included. This has now been added in the Methods section to state that *All HCPs involved in the CATHETER II trial were given information about the qualitative component and invited to take part in focus groups or for interview and all interested HCPs were included.***
7. RESULTS **– the quotes selected are very short in nature**. This perhaps reflects the surface and superficial nature of the data collection. More in-depth and rich quotes would be preferred.	***Short quotes were used in the Results section to manage the length of the article. We have now included some more in-depth quotes, and further quotations are presented as Supplementary Table.***
8. How was data saturation known to be achieved?	***Due to the information power approach to the sampling (as describe above) and theory based analytic approach we focused on how well our codes characterise the dataset as an indication of when further data is unlikely to give further information (*Guest G et al 2020, & Francis JJ et al 2009*). To operationalise this approach, we first analysed 6 interviews in each category (first 18 interviews) and coded the data. We then apply conception of 'no new information' on any of the TDF/TFA or inductive codes' after each third new interview and following that each new interview in each category. Time 2 interviews were conducted with 30 available participants from time 1. There were 10 participants who were unable to attend the time 2 interviews therefore further 10 interviews were conducted with participants with similar characteristics as far as possible.***<br><br>***Due to the small cohort of HCPs in the trial who consented to take part in the qualitative study there was only one focus group conducted, and one further HCP interview was conducted to check the data and ensure the robustness of the focus group data. The participants' views within the focus group and the following HCP interview were consistent and no new information emerged from the interview therefore no further interviews were conducted.***
9. Thanks for including your interview guide. The question route is very exploratory around the trial, however, there appears to be very limited questioning on translation to routine care, understanding of implementation factors and barriers, facilitators, and sustainability in routine practice. (e.g economic impact to the	***In line with the qualitative study design the topic guides were exploratory around the trial to focus on the specific objectives of this embedded qualitative study.* These were developed following discussions with the study team, review of the scientific literature and existing patient interviews on www.healthtalk.org and were piloted with public and patient representatives and refined. *The topic guides were used to ensure consistency in data collection. However, Interviews and focus group were semi-structured with open ended questions,***

health system and patients, for example). The question guide seems very surface in nature.	***participants were encouraged to feel free to express their views on issues that they felt important beyond the topic guide as well resulting in robust data on both patients and health care providers perspectives on both receipt and provision of care.***<br><br>***Economic implication of the washout polices on the health system was investigated separately in the CATHETER II trial and was not the focus of the qualitative study.***
10. **Caution with terminology such as 'demonstrated'** in qualitative research – See ") this qualitative study demonstrated that the catheter calendar and monthly phone calls were a key component of the CATHETER II trial and might have contributed to participant retention and may also have important clinical translational considerations for optimising patient care and support" – the aim of qualitative research is primarily to explore, and not demonstrate.	***The term 'demonstrated' has now been replaced with appropriate terms.***
11. **There is no 'Limitations' section** – the study limitations are not acknowledged.<br><br>Bladder washouts may need to be considered carefully or contraindicated for those who are at risk of developing autonomic dysreflexia, or patients with neurogenic bladder conditions.<br><br>Other aspects such as strategies for minimising the risk of infection while flushing the catheter, precautions for those who experience bladder spasms, and small compliance bladder may need to be explored further.<br><br>Introducing the catheter flushes as a prophylactic measure may not eliminate the root cause of the blockage. Consider exploring the contributing factors of catheter blockages and how to address them.	***The limitation subheading has now been added to the 'strengths and limitations' section.***<br><br>***We agree with the reviewer about including participant with who are* at risk of developing autonomic dysreflexia, or patients with neurogenic bladder conditions. Please note that *patients with risk for autonomic dysreflexia (Spinal Injury above or at T6) were excluded in the CATHETER II trial.***<br><br>***We also agree* that strategies for minimising the risk of infection while flushing the catheter for those who experience bladder spasms, and small compliance bladder may need to be explored further. This is outside the scope of this qualitative study. This would be a quantitative measure as due to small sample size, sub-group analysis was not performed in the clinical paper (*Reference 12- Abdel-Fattah et al., 2024*). This can be explored by using real world evidence (Big Data).**<br><br>**We agree, exploring the contributing factors of catheter blockages in important. However, this outside the scope of this qualitative study. This can be explored by using real world evidence (Big Data).**

VERSION 2 - REVIEW

Reviewer 2

Name Choudhary, Sidhya

Affiliation Government Medical College and Hospital

Date **27-Feb-2025**

COI

The study offers valuable insights into prophylactic catheter washout but has some key limitations:

1. The findings are based on participants already engaged in the trial, leaving out perspectives from those less inclined to adopt washout. Exploring barriers to engagement among less motivated patients would add depth.

2. The number of centers (15 or 21) is inconsistent and needs clarification.

3. Lack of Standardized Guidelines: While the study highlights a gap in guidance, it does not suggest clear pathways for policy development.

4. Confidentiality Concerns: Patient names should be removed from Table 1 to maintain privacy.

5. The study does not fully consider differences in patient responses, such as variations due to age, comorbidities, or prior catheter-related issues.

6. Missing Cost-Effectiveness Analysis: There is no discussion on whether prophylactic washout is financially viable in the long run.

7. Retention Factors Overlooked: The study credits retention to the catheter calendar and phone calls but does not examine other possible influencing factors.

8. Repetitive Discussion and no need to mention patient dialogues: A more concise approach would improve readability.

VERSION 2 - AUTHOR RESPONSE

Reviewer 2 Comments to the Author:<br>The study offers valuable insights into prophylactic catheter washout but has some key limitations:<br><br>1. The findings are based on participants already engaged in the trial, leaving out perspectives from those less inclined to adopt washout. Exploring barriers to engagement among less motivated patients would add depth	The authors have discussed this. As the study is now complete, no further recruitment and data collection from additional participants is possible. Nonetheless, we acknowledge this as a limitation in the Discussion section as follows:<br><br>*“It is important note that the findings from this study need to be considered in light of small sample size of HCPs and that the patients in the study were the ones already engaged in the CATHETER II trial. Therefore, their views may not be representative of those who are less*

	inclined to engage in washout therefore did not take part in the CATHETER II study."
2. The number of centres (15 or 21) is inconsistent and needs clarification.	Participants for this qualitative study were recruited from 16 out of the 21 Catheter II trial sites. HCPs were recruited from 6 out of the 21 Catheter II trial sites.<br><br>This has now been clarified in page 8 and 11 by stating that<br><br>*"Focus group and interviews were conducted with 7 HCPs at least 6 months after the study commenced. Focus group was conducted with health care professionals involved in the trial from 6 of the 21 sites"*<br><br>"...trial participants aged 23 to 100 years from 16 out of the 21 CATHETER II recruitment sites across the UK were included"
3. Lack of Standardized Guidelines: While the study highlights a gap in guidance, it does not suggest clear pathways for policy development.	We have addressed this by firmly stating in the Discussion the following: *"We believe that this study, especially the outcomes on acceptability, feasibility and effectiveness of washout training and weekly prophylactic washout on a behavioural level, could inform development of catheter washout policies that may ease burdens on healthcare providers."*
4. Confidentiality Concerns: Patient names should be removed from Table 1 to maintain privacy.	These are Pseudonyms and not real patient names.
5. The study does not fully consider differences in patient responses, such as variations due to age, comorbidities, or prior catheter-related issues.	Notable differences (or lack thereof) in patients' responses in relation to gender, age, prior catheter related issues have been considered in all relevant findings (page 14, 17, 19 and 22), for example by stating that:

	"There were no notable differences in participants' descriptions of their prior LTC experience, attitude, expectation, and potential barriers and facilitators to their participation between different groups (e.g. arm of trial, gender, or age)"<br><br>Comparison of participants' responses based on co-morbidities was outside the scope of this study.
6. Missing Cost-Effectiveness Analysis: There is no discussion on whether prophylactic washout is financially viable in the long run.	Economic implication of the washout polices on the health system was investigated separately in the CATHETER II trial and was not the focus of this qualitative study.
7. Retention Factors Overlooked: The study credits retention to the catheter calendar and phone calls but does not examine other possible influencing factors.	The study only suggests (based on high participant retention between T1 and T2 within the qualitative study) that the catheter calendar and monthly phone calls might have contributed to participant retention. However, as we did not directly assess retention strategies, examining the the influence of other factors was not within the scope of this study.<br><br>This has been clarified in page 23 by stating that:<br><br>"This qualitative study suggested that the catheter calendar and monthly phone calls were a key component of the CATHETER II trial and **might have contributed** to participant retention and may also have important clinical translational considerations for optimising patient care and support. Although we did not directly assess retention strategies, participant retention was high between T1 and T2 within this qualitative study."
8. Repetitive Discussion and no need to mention patient dialogues: A more concise approach would improve readability.	The paper has been revised by authors to address any repetitive discussion. While there are some issues discussed

	multiple times, they were deemed necessary by authors in order to address distinct implications of the findings in the study. For example, the washout training has been discussed in terms of its feasibility, and again in relation to it being essential and/or useful. We have rephrased some sentences in the Discussion to avoid repetitiveness and ensure clarity.<br><br>The authors discussed the suitability of using participants' quotations and all agreed that this is standard practice in reporting results of qualitative research, where representative quotations from the participants are important to rigorously illustrate the findings, demonstrate how the findings and interpretations have arisen and ensure that findings are grounded in the data.<br><br>Use of quotations in this paper also conforms to the standard practice in line with BMJ guidelines for reporting qualitative research (e.g. Consolidated criteria for Reporting Qualitative research, COREQ (Tong et al., 2007), which also highlights the effective application of quotations.)<br><br>We want to draw the reviewer's and editor's attention to the use of this standard practice in the following publications as examples:<br>1. Merriam, S. B., & Tisdell, E. J. (2016). Qualitative Research: A Guide to Design and Implementation (4th ed.). San Francisco, CA: Jossey-Bass.<br>2. Corden, A., & Sainsbury, R. (2006). Exploring 'Quality': Research Participants' Perspectives on Verbatim Quotations. International Journal of Social Research Methodology: Theory & Practice, 9(2), 97–110<br>3. Braun V, Clarke V Using thematic analysis in qualitative research.

	Qualitative Research in Psychology 3 (2), 77-101.
